# Evidence of effective delivery of the human papillomavirus (HPV) vaccine through a publicly funded, school-based program: the Ontario Grade 8 HPV Vaccine Cohort Study

**DOI:** 10.1186/1471-2458-14-1029

**Published:** 2014-10-03

**Authors:** W Ting Lim, Kim Sears, Leah M Smith, Guoyuan Liu, Linda E Lévesque

**Affiliations:** Department of Public Health Sciences, Queen’s University, Kingston, Ontario K7L 3N6 Canada; Institute for Clinical Evaluative Sciences, Queen’s University, Kingston, Ontario K7L 3N6 Canada; School of Nursing, Queen’s University, Kingston, K7L 3N6 Canada; Department of Epidemiology, Biostatistics and Occupational Health, McGill University, Montréal, H3A 1A2 Canada

**Keywords:** Human papillomavirus vaccines, HPV vaccine, Series completion, Adherence, Compliance, Cohort study

## Abstract

**Background:**

Proper administration of the human papillomavirus (HPV) vaccine (three doses at 0, 2, and 6 months) will likely influence the vaccine’s effectiveness and the impact of vaccination programs on health outcomes. Therefore, we assessed HPV vaccine series completion and on-time dosing in Canada’s largest publicly funded, school-based HPV vaccination program.

**Methods:**

Using administrative health and immunization databases, we identified a population-based cohort of girls eligible for Ontario’s Grade 8 HPV vaccination program in the 2007/08-2009/10 program years who received at least one dose of the vaccine. We determined the number of doses received and calculated the percentage of girls that completed the three-dose series in Grade 8 and Grades 8–9. To assess on-time dosing, the number of days between doses 1–2, 2–3, and 1–3 was calculated and categorized (e.g., too short, on schedule, too long) based on the manufacturer’s recommendations. Analyses were also stratified by program year.

**Results:**

We identified a cohort of 55,798 girls who initiated the vaccination series. Series completion was high in the Grade 8 window (81.8%) and increased by approximately 6% in Grade 9. Series completion was similar across the three program years. 70.8%, 98.5%, and 86.1% of girls were classified as ‘on schedule’ for dosing intervals 1–2, 2–3, and 1–3, respectively; 70.0% of girls received all three doses in perfect accordance with dosing recommendations. Stratification revealed that on-time dosing was highest in the first two years of the program (85.6% and 80.6%), but dropped to 42.1% in the 2009/10 year when H1N1 vaccination programs were prioritized.

**Conclusions:**

Our study demonstrates that delivery of the HPV vaccine through a free, school-based program is an effective method of ensuring high completion and on-time dosing, but may not be sufficient to guarantee high coverage.

## Background

Gardasil^®^ is a quadrivalent human papillomavirus (qHPV) vaccine designed to protect against infection with four types of HPV that cause 90% of anogenital warts (HPV-6 and -11) and 70% of cervical cancers (HPV-16 and -18) [1–3]. The largest randomized controlled trial (RCT) of the qHPV vaccine showed it to be highly efficacious in preventing vaccine-type-specific anogenital warts (99%; 95% CI 96-100%) and pre-cancerous cervical lesions (96%; 95% CI 91-98%) [4]; other RCTs reported similar levels of efficacy [5, 6]. Importantly, these promising results were established following adherence and compliance to the recommended three-dose vaccine schedule of 0, 2 and 6 months. Although the qHPV vaccine has now been commercially available for over six years, there is limited information on whether individuals outside of clinical trials have been following the recommended dosing schedule. Since the number *and* timing of doses will likely influence the vaccine’s effectiveness and the impact of vaccination programs on adolescent health outcomes, we undertook a population-based retrospective cohort study to evaluate HPV vaccine series completion (adherence) and on-time dosing (compliance) among Grade 8 girls in a publicly funded, school-based HPV vaccination program in Ontario, Canada.

## Methods

### Ontario’s Grade 8 HPV vaccination program

Soon after the qHPV vaccine was licensed for use in Canada, the National Advisory Committee on Immunization (NACI) released a statement recommending routine immunization for all girls aged 9–13 years [7]. Based on these recommendations, the Canadian government announced in 2007 that they would be investing $300 million toward the launch of a national qHPV vaccination program aimed at immunizing young girls, ideally before the onset of sexual activity. Since this money was allocated to provinces and territories on a per-capita basis, the Ontario government received $117 million to design and implement Ontario’s Grade 8 HPV vaccination program [8]. This program, which began in September of 2007, offers three doses of the qHPV vaccine free of charge to all Grade 8 girls in the province. The program is coordinated by the province’s 36 health units (i.e., administrative health regions), primarily through school-based immunization clinics. As a result, doses are generally administered by public health nurses in schools in September/October, November/December, and March/April of each school year to correspond with the recommended 0-, 2-, 6-month dosing schedule of the vaccine. While the vast majority of publicly funded HPV vaccine doses are administered through the school clinics, eligible girls also have the option of obtaining the vaccine at their health unit or through their family physician at no cost. Girls have until the end of their Grade 8 year to initiate the vaccination series (Grade 8 eligibility period) and until the end of their Grade 9 year to complete it (program eligibility period). Approximately 84,000 girls are eligible for Ontario’s Grade 8 HPV vaccination program each year, but HPV vaccination is optional [9].

### Data sources

Two administrative health databases were used for this study – the Registered Persons Database (RPDB) and the Immunization Record Information System (IRIS) database. The RPDB is the population registry of insured persons for Ontario’s universal healthcare programs. It has been used extensively in health research, including in the post-marketing evaluation of drugs and vaccines [10–12]. In this study, the RPDB was used for information on the socio-demographics of cohort members. A copy of the RPDB is housed at the Institute for Clinical Evaluative Sciences (ICES).

Information on mandatory and optional vaccinations, including qHPV vaccination, was obtained using the Immunization Record Information System (IRIS) database. Developed by the Ministry of Health and Long-Term Care, IRIS databases are maintained by each of Ontario’s 36 health units to track and record the vaccinations of all school-aged children in their jurisdiction. As a result, the province’s 36 IRIS databases contain individual-level information on all qHPV vaccine doses administered through the publicly funded program, regardless of whether the vaccine is administered at school, a physician’s office, or the public health unit. Although IRIS may not capture all HPV vaccine doses of eligible girls who chose to receive the vaccine outside of the publicly funded program, given the high cost of the otherwise free vaccine (approximately $450CAD for three doses), this is unlikely to have an important impact on the completeness of the IRIS data. To investigate the validity of the HPV vaccine data contained in IRIS, a validation study was executed in one of Ontario’s health units (Kingston, Frontenac, Lennox, and Addington; KFLA) using paper vaccination records as the gold standard. The validation study demonstrated that the database captured individuals’ HPV vaccination status with high sensitivity (99.8%; 95% CI: 99.3-99.9) and specificity (97.7%; 95% CI: 96.3-98.7); it also demonstrated that 98.6% of vaccination dates were accurate [13]. At the time of the current study an encrypted copy of the IRIS database of 21 health units had been transferred to ICES and were available for use in this study. In the IRIS and RPDB databases housed at ICES, residents of Ontario are represented by a unique encrypted identifier, enabling complete record linkage at the level of the individual across databases and time. In Ontario, the transfer, linkage, and encryption of health data is permitted under section 45.1 of the Personal Health Information Protection Act (2004) and was executed under data sharing agreements between each participating health unit and ICES. Although all 36 health units were recruited for this study at the same time, due to various administrative delays, the data for the remaining 15 health units were not yet available.

### Study population

The source population for this study was the Ontario Grade 8 HPV Vaccine Cohort, which is comprised of all girls eligible for the province’s publicly funded HPV vaccination program between 2007 and 2011. Members of the source cohort were identified using the RPDB and IRIS databases. Since school grade is not available in these databases, the cohort was identified using birth year because girls are typically 13 years of age by December 31 of their Grade 8 year. Therefore, the source cohort consisted of all girls born in 1994, 1995, 1996, and 1997 in Grade 8 in Ontario in the 2007/08, 2008/09, 2009/10, and 2010/11 school years, respectively. Although this method of cohort identification may miss girls who were advanced or held back a school grade, the validation study on the KFL&A IRIS database demonstrated that this cohort definition correctly identified 96.4% of eligible girls [14]. Cohort members were followed from September 1 of their Grade 8 year (cohort entry) until the first of: date of death or date of study end (March 31, 2011). For the purposes of the current study, the source cohort was restricted to girls for whom complete IRIS data were available. As a result, the study cohort included girls who resided within 21 of the participating health units during 2007/08-2009/10 program years (i.e., 1994–1996 birth cohorts); the 1997 birth cohort was excluded because girls had not completed their Grade 8 year by the study end date. Subsequently, IRIS was used to further restrict the cohort to girls who received at least one dose of the qHPV vaccine during study follow up. Girls who received a dose prior to cohort entry were excluded. After record linking the study cohort with the IRIS database, we discovered that a small number of cohort members had duplicate qHPV vaccination records with discordant vaccination dates (n = 9). Most often, such duplicate records arise when a girl moves from one health unit to another and the parental self-report of vaccination status does not correspond with the electronic record. These girls were excluded from the study cohort because the correct vaccination date could not be readily identified.

### HPV vaccination status and dose timing

Information on the qHPV vaccination history (e.g., date of administration) of cohort members was ascertained using the IRIS database. To describe HPV vaccine doses, the number of doses received by each cohort member was determined. To assess vaccine series completion (adherence), cohort members were categorized based on whether or not they received the three recommended doses of the vaccine (1–2 doses vs. ≥3 doses) in the Grade 8 eligibility period (i.e., September 1 until August 31 of the girl’s Grade 8 year) and in the program eligibility period (i.e., September 1 of Grade 8 until August 31 of Grade 9); in accordance with the program guidelines, girls had to have initiated the vaccination series in Grade 8 in order to be classified as adherers. To assess on-time dosing (compliance) with the recommended 0-, 2-, 6-month schedule, the number of days between doses (1–2, 2–3, 1–3) was calculated. Each dosing interval was subsequently classified according to how it corresponded with the recommended dosing interval (e.g., too short, on schedule, too long). These classifications were created based on the dosing schedule (and ‘flexibility period’) specified in the Gardasil^®^ product monograph [1]. Specifically, the interval between dose one and two was categorized as: (i) too short (<30 days), (ii) on schedule (30–90 days), or (iii) too long (>90 days); the interval between dose two and three was dichotomized based on whether it met the minimum recommended interval of at least three months (<90 days vs. ≥90 days); and the interval between dose one and three (time to series completion) was classified as: (i) too short (interval one to three <120 days *or* interval two to three <90 days), (ii) on schedule (interval one to three 120–240 days *and* interval two to three ≥90 days), (iii) acceptable (interval one to three 241–365 days), or (iv) too long (interval one to three >365 days). The ‘acceptable’ category was created to address the fact that the product monograph stipulates that all three doses should be given within a one-year period and that the third dose should be given by Month 8 (Day 240). Finally, on-time dosing was dichotomized based on whether girls received all three doses according to the recommendations (i.e., all intervals ‘on schedule’). On-time dosing was calculated overall as well as by program year.

### Baseline characteristics

The RPDB and IRIS databases were used to ascertain baseline information on the socio-demographics and vaccination history of cohort members.

The socio-demographic factors considered included age, place of residence, and neighbourhood income quintile at cohort entry. Place of residence and neighbourhood income were obtained by linking a girl’s postal code at cohort entry with the 2006 Canadian Census data. Neighbourhood incomes were categorized into provincial quintiles, and place of residence was dichotomized as urban (≥10,000 persons) or rural (<10,000 persons) [15].

IRIS was used to ascertain information on vaccines received prior to cohort entry. In particular, IRIS was used to determine whether cohort members had received the measles-mumps-rubella (MMR) and diphtheria-tetanus-polio (DTP) vaccines (which are considered mandatory under the *Immunization of School Pupils Act*, *1982* [16]), as well as the hepatitis B and meningococcal conjugate vaccines (which are optional vaccines offered to all Grade 7 students in Ontario through school-based immunization clinics). Previous vaccination was defined based on receipt of at least one dose of the vaccine of interest anytime prior to cohort entry.

### Statistical analysis

Means and proportions of continuous and categorical variables were calculated to describe the baseline characteristics of the study cohort.

To describe qHPV vaccine use, the proportion of cohort members who received one, two, three, or greater than three doses was calculated. To determine vaccine series completion, we calculated the proportion of cohort members who completed the vaccination program in the Grade 8 eligibility period and the proportion of cohort members who completed the vaccination series during the program eligibility period. These analyses were performed overall as well as by program year.

To assess dose intervals, we determined the number of days between Dose 1 and Dose 2 (among girls who received at least two doses), Dose 2 and Dose 3, and Dose 1 and Dose 3 (among girls who received at least three doses). Univariate statistics were used to describe these intervals. As previously described, dose timing was categorized on the basis of compliance with the recommended vaccine schedule; proportions were used to describe these measures of compliance.

P-values were calculated to assess significant changes in use across program year and eligibility period. Chi-square tests were used when observations were independent (i.e., across program year) and McNemar’s tests were used when observations were not independent (i.e., across eligibility period).

All statistical analyses were performed using Statistical Analysis Software (SAS) version 9.2 (SAS Institute Inc., Cary, NC).

### Ethics

This study was approved by Queen’s University’s Health Sciences Research Ethics Board and Sunnybrook Health Sciences Centre’s Ethics Review Board.

## Results

**Figure 1:**
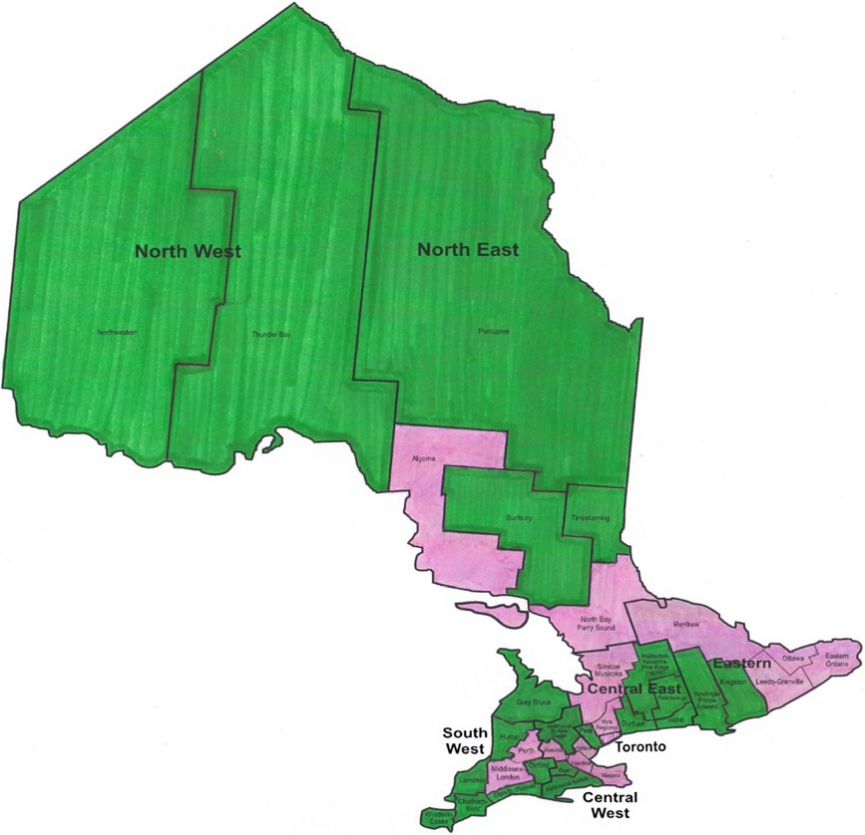
**Geographic representation of participating health units.** The green regions represent health units whose immunization records were available at the time of the analysis

Within the 21 health units for which data were available, 111,798 girls were eligible for publicly funded HPV vaccination in the 2007/09 to 2009/10 school years. These girls represented approximately 40% of all girls eligible for publicly funded qHPV vaccination in Ontario; geographically, they were representative of all girls eligible for publicly funded qHPV vaccination in Ontario (Figure 1). Approximately 50% (N = 55,798) of girls within our study sample initiated the HPV vaccination series during the study period and comprised our study cohort. Cohort members were a mean age of 13.2 years at cohort entry and were followed for an average of 2.6 years (Table 1). Most cohort members (84.5%) resided in an urban setting and approximately one third resided in lower-income neighborhoods. Almost all cohort members had received the MMR and DTP vaccines, and most had received the optional hepatitis B and meningococcal conjugate vaccines; 73.8% had received all four vaccines.

**Figure 2:**
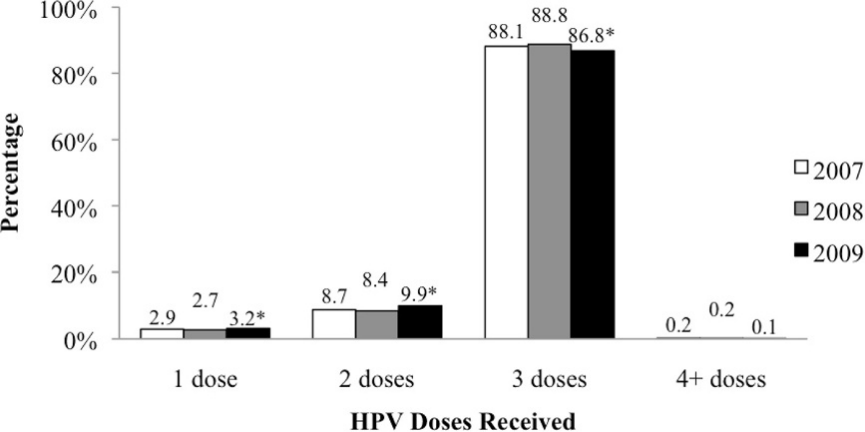
**Number of doses received during the program eligibility period,** **stratified by program year.** *Proportion significantly different from previous program year (p<0.05).

Figure 2 depicts the number of HPV vaccine doses received by cohort members during their Grade 8 and 9 school years. While the number of doses received in the 2009/10 year was statistically significantly different than those in the previous program year, the absolute differences were small. Only 0.15% of girls received more than the recommended three doses.

**Table 1:** **Baseline characteristics of study cohort** (**N** = **55**,**798**)

Characteristic	n (%)
**Socio**-**demographics**	
*Age* (*years*), *mean* (*SD*)	13.2 (0.3)
*Place of residence*	
Urban	47,130 (84.5)
Rural	8,630 (15.5)
Missing	38 (0.1)
*Income Quintile*	
1 (low)	8,054 (14.4)
2	10,428 (18.7)
3	12,648 (22.7)
4	12,767 (22.9)
5 (high)	11,698 (21.0)
Missing	203 (0.4)
**Vaccination History**	
*Mandatory Vaccines*	55,094 (98.7)
Measles, mumps, rubella (MMR)	55,272 (99.1)
Diphtheria, tetanus, pertussis (DTP)	55,316 (99.1)
*Optional Vaccines*	41,505 (74.4)
Hepatitis B	48,393 (86.7)
Meningococcal conjugate	44,181 (79.2)
*Mandatory and optional vaccines*	41,158 (73.8)

**Figure 3:**
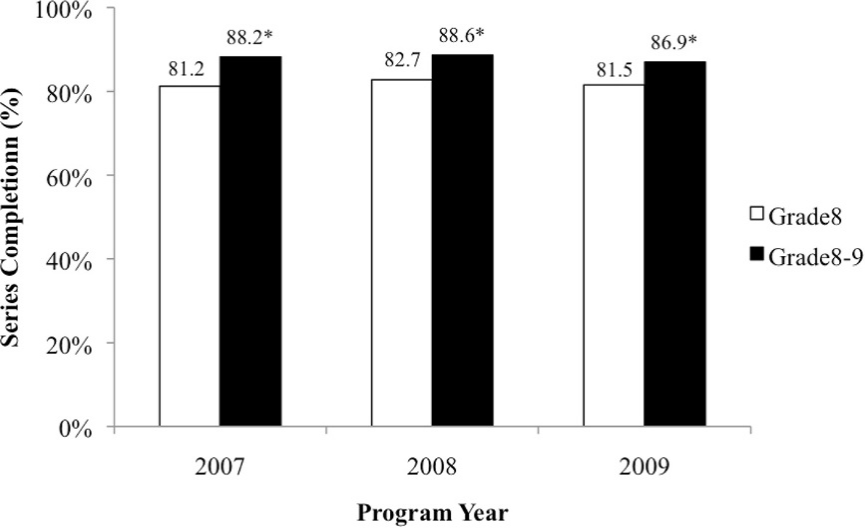
**Completion of the three**-**dose series across program year,** **according to eligibility period.** *Proportion in program eligibility period (i.e., Grade 8-9) significantly different from proportion in Grade 8 eligibility period (p<0.0001).

Series completion was high during the Grade 8 eligibility period (81.8%) and increased by approximately 6% in Grade 9 (Figure 3). Few cohort members (*n* = 136) initiated the vaccination series after the program eligibility period (data not shown).

**Figure 4:**
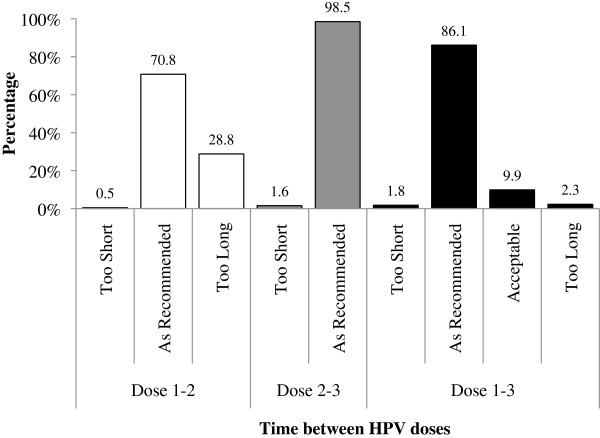
**Compliance with recommended dosing intervals.**

On average, the time between doses one and two, two and three, and one and three was 2.8 months (standard deviation [SD] = 1.5), 4.2 months (SD = 1.6), and 6.9 months (SD = 2.0), respectively. Figure 4 describes compliance with the recommended dosing intervals. Time to series completion (Dose 1–3) was good, with 86.1% girls receiving their third dose in perfect accordance with the recommendation and an additional 9.9% completing their series within one year. A small percentage of doses were received earlier than recommended (<2.0%). Although only 70.8% of girls received their second dose within the recommended time frame, Table 2 (which stratifies compliance by program year) demonstrates that on-time receipt was high in the first two program years, but dropped to 43.5% in 2009/10. In accordance with this drop in Year 3, overall, only 70.0% received all doses of the vaccine on schedule, despite on-time dosing of 86% and 81% in the first two years.

**Table 2:** **Compliance by program year**

Compliance (%)	Program year(s)				p-value*	
	2007/08	2008/09	2009/10	2007/08 - 2009/10	2007/08 & 2008/09	2008/09 & 2009/10
**Dose 1-** **2**						
*Short*	0.2	0.2	1.0	0.5	<0.0001	<0.0001
*On schedule*	85.7	82.1	43.5	70.8		
*Long*	14.2	17.6	55.4	20.8		
**Dose 2-** **3**						
*Short*	1.2	0.5	3.2	1.6	<0.0001	<0.0001
*On schedule*	98.8	99.5	96.8	98.4		
**Dose 1-** **3**						
*Short*	1.2	0.5	3.9	1.8	<0.0001	<0.0001
*On schedule*	89.8	89.0	79.1	86.1		
*Acceptable*	7.4	7.2	15.4	9.9		
*Long*	1.7	3.4	1.6	2.3		
**Overall**						
*All on schedule*	85.6	80.6	42.1	70.0	<0.0001	<0.0001
*Not on schedule*	14.4	19.4	57.9	30.0		

*based on Chi-square tests.

## Discussion

Using population-based data from Canada’s most populous province, Ontario, we found that completion of the HPV vaccine’s three-dose series through a publicly funded, school-based program was high. Moreover, it was consistently high across the first three years that the program was offered. Generally, compliance with the recommended timing of the dosing schedule was also good, but there were delays in the administration of Dose 2 in the 2009/10 program year.

Almost 90% of cohort members who initiated the HPV vaccination series went on to receive all three recommended doses of the vaccine. Not only is this number impressive in its own right, but it also represents one of the highest levels of series completion that has been reported to date [17–23]. Moreover, if, as suggested by recent studies [24, 25], two doses of the HPV vaccine provide as much protection as the full series, then over 95% of our study population would be considered fully immunized. Within this group, a small percentage is documented as having received more than three doses of the vaccine. While some of these cases may be the result of documentation errors or erroneous self-reports, excess vaccination has been previously reported in situations where multiple doses are involved [26]; therefore, the clinical impact of such potential medical errors must be investigated.

To our knowledge, this is the first study to assess series completion and on-time dosing of quadrivalent HPV vaccine within the context of a publicly funded, school-based program. However, there have been some reports on compliance when the vaccine is accessed through private means. For example, Tan et al. [27] reported on series completion and on-time dosing of qHPV vaccine doses in North Carolina. They found that, among the 138,823 females who initiated the vaccination series, only 55% completed the series and 28% completed it on time. Furthermore, they found that both completion and on-time dosing decreased dramatically between 2006 and 2009. Similarly, Dorell et al. [28] reported on compliance with the recommended HPV vaccine-dosing intervals among a nationally representative sample of girls aged 13–17 years in the United States. They found that almost half of girls who completed the vaccination series did so in a period longer than the recommended interval. In contrast, our findings showed that 70% of girls completed each aspect of the dosing regimen in perfect accordance with the recommended dose-timing schedule. We also found that usage was fairly consistent across program years. The fact that series completion and on-time dosing were so much greater in our study population likely reflects the value of a well-run publicly funded program delivered through school-based clinics, where a student need only be present at school to receive her next scheduled dose of the vaccine. Nevertheless, as HPV vaccine use was not perfect in our study population, the predictors of both series completion and on-time dosing should be investigated to further improve proper receipt of this vaccine.

Despite the promising results presented here, it is important to note that more than half of girls eligible for free HPV vaccination in the 2009/10 program year received their second dose later than recommended. These results are not surprising given the H1N1 outbreak and corresponding immunization campaign that began in the fall of 2009 that was, understandably, prioritized over HPV vaccination programs. Another possible explanation for the delays in receipt may be increased school absenteeism due to illness during that time, as was believed to be the case in Scotland’s school-based program [22]. Regardless of the reason, the delays in administration of Dose 2 may have implications in terms of vaccine effectiveness for the girls affected. Along the same lines, we also found that a small percentage of girls received doses early. Although there is evidence suggesting that receiving doses using alternative schedules does not have a major impact on immunogenicity, [29] the clinical implications of receiving doses outside of the indicated dosing intervals should be further investigated.

Although series completion and on-time dosing were high in this study population, this must be considered in the context of fairly low HPV vaccine series initiation. Indeed, only 50% of girls eligible for Ontario’s Grade 8 HPV vaccination program opted to receive the initial dose of the vaccine, meaning only 42% of all eligible girls would be regarded as fully immunized. Unfortunately, despite high HPV vaccine series completion and on-time dosing, such low coverage suggests this vaccination program may have a considerably smaller impact on the burden of disease in the population than anticipated. To address this issue, Ontario and other programs struggling with low HPV vaccine series initiation should draw on the strategies implemented by regions that have demonstrated success, such as Scotland. In particular, Sinka et al. [22] reported that, following the targeted efforts made in Scotland to maximize coverage of the bivalent HPV vaccine [30] more than 90% of all girls eligible for the country’s publicly funded, school-based program received the first dose of the vaccine and more than 80% of eligible girls received all three doses.

A potential limitation of our study is that if a girl moved to a location outside of the 21 health units for which immunization data were available her immunization data may be incomplete. Consequently, our study may have underestimated series completion. In addition, we did not have complete follow-up time on girls eligible for the 2009/10 program year since their immunization data were truncated at March 31 of their Grade 9 year. Nevertheless, the consistency in series completion across program years suggests that few girls receive doses after that time. Another potential source of misclassification is inaccurate recording of vaccination dates. However, a recent re-abstraction in one of the health units found that 98.6% of HPV vaccination dates were accurate in IRIS [13]. Due to the standardized procedures in place for documenting immunizations of school-aged children in Ontario, we expect the high validity of these results to be generalizable to other IRIS databases. As a result, we expect the impact of any misclassification of dates to be minimal. A final limitation of our study is that the results may not be generalizable to areas with different methods of HPV vaccine program delivery. We would however expect them to be generalizable to other jurisdictions that provide the quadrivalent HPV vaccination series to young girls through free, school-based clinics, such as those that exist across Canada.

## Conclusions

Publicly funded, school-based HPV immunization programs overcome financial and accessibility barriers to healthcare, thereby creating an ideal setting in which vaccine series completion and on-time dosing can be optimized. Indeed, the results of our study suggest this approach to program delivery is enabling the vast majority of girls who initiate the series to complete it according to the recommended schedule. Nevertheless, our findings also indicate that removing financial and accessibility barriers alone may not be sufficient for ensuring high HPV vaccine coverage. Future studies should identify other barriers to HPV vaccine series initiation and investigate the efficacy and safety of the HPV vaccine when received outside of the recommended dosing scheduling.
